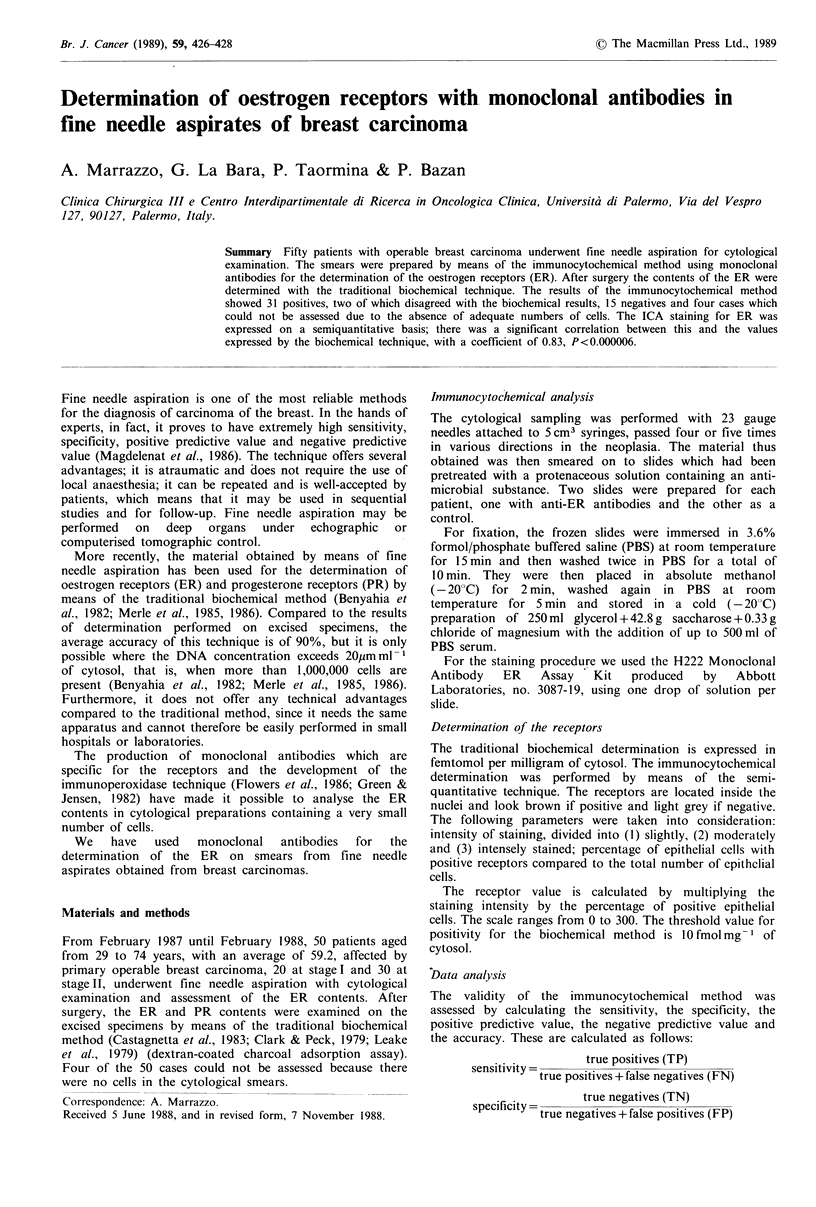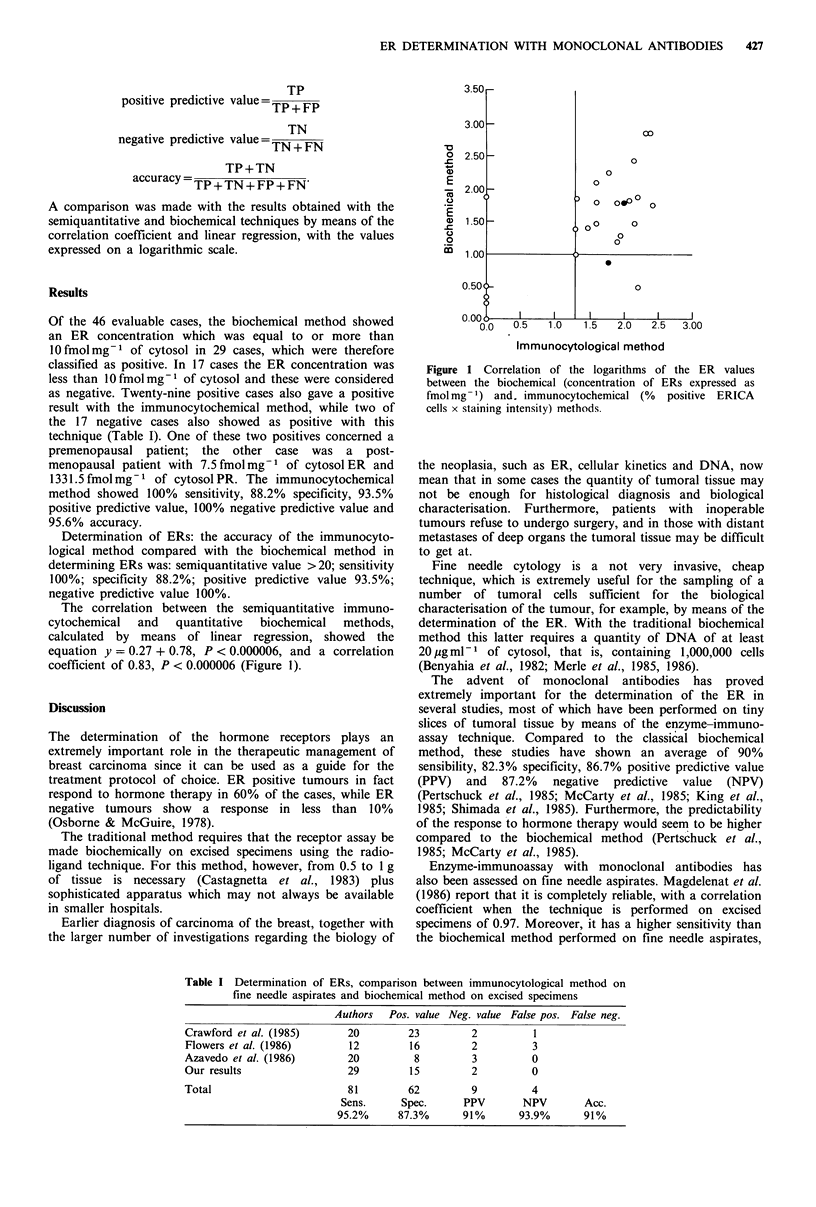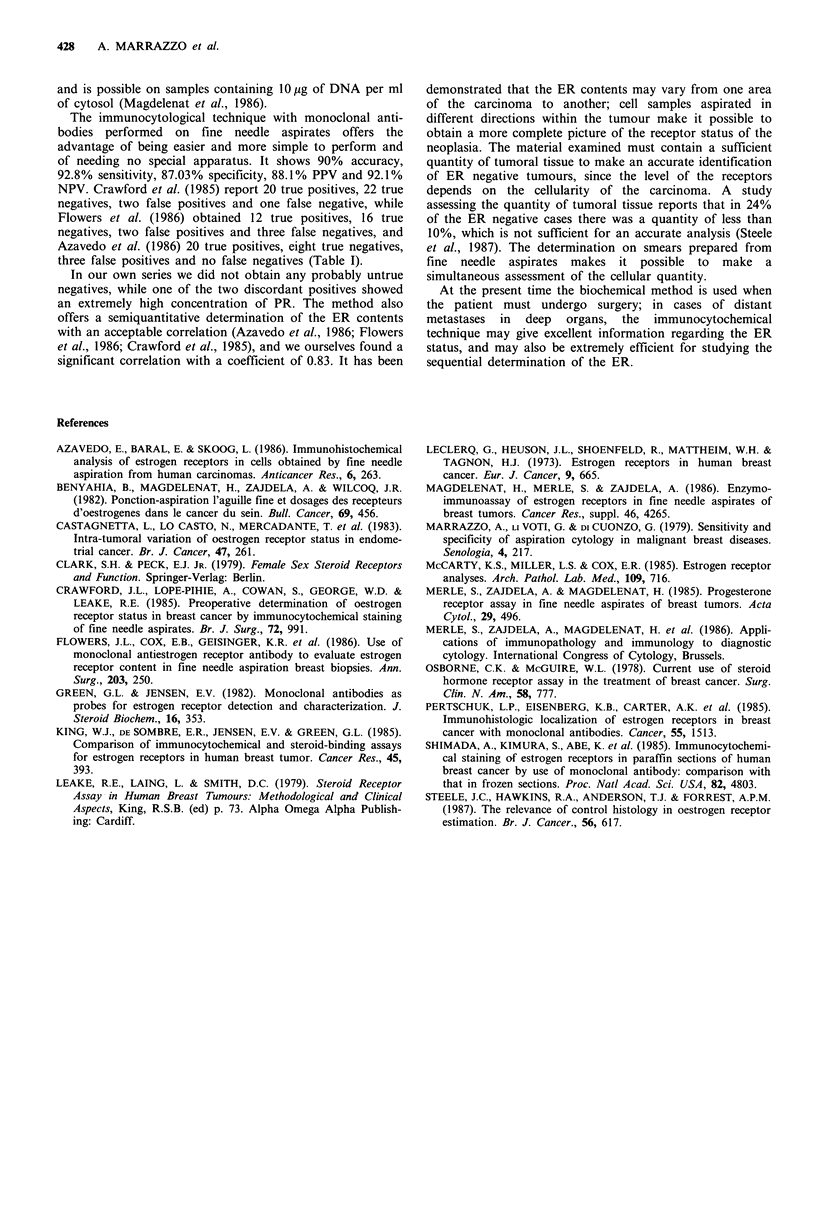# Determination of oestrogen receptors with monoclonal antibodies in fine needle aspirates of breast carcinoma.

**DOI:** 10.1038/bjc.1989.86

**Published:** 1989-03

**Authors:** A. Marrazzo, G. La Bara, P. Taormina, P. Bazan

**Affiliations:** Clinica Chirurgica III e Centro Interdipartimentale di Ricerca in Oncologica Clinica, UniversitÃ di Palermo, Italy.

## Abstract

Fifty patients with operable breast carcinoma underwent fine needle aspiration for cytological examination. The smears were prepared by means of the immunocytochemical method using monoclonal antibodies for the determination of the oestrogen receptors (ER). After surgery the contents of the ER were determined with the traditional biochemical technique. The results of the immunocytochemical method showed 31 positives, two of which disagreed with the biochemical results, 15 negatives and four cases which could not be assessed due to the absence of adequate numbers of cells. The ICA staining for ER was expressed on a semiquantitative basis; there was a significant correlation between this and the values expressed by the biochemical technique, with a coefficient of 0.83, P less than 0.000006.


Br. J. Cancer (1989), 59, 426–428 © The Macmillan Press Ltd., 1989

### Determination of oestrogen receptors with monoclonal antibodies in fine needle aspirates of breast carcinoma

A. Marrazzo, G. La Bara, P. Taormina & P. Bazan

*Clinica Chirurgica III e Centro Interdipartimentale di Ricerca in Oncologica Clinica, Università di Palermo, Via del Vespro 127, 90127, Palermo, Italy.*

**Summary** Fifty patients with operable breast carcinoma underwent fine needle aspiration for cytological examination. The smears were prepared by means of the immunocytochemical method using monoclonal antibodies for the determination of the oestrogen receptors (ER). After surgery the contents of the ER were determined with the traditional biochemical technique. The results of the immunocytochemical method showed 31 positives, two of which disagreed with the biochemical results, 15 negatives and four cases which could not be assessed due to the absence of adequate numbers of cells. The ICA staining for ER was expressed on a semiquantitative basis; there was a significant correlation between this and the values expressed by the biochemical technique, with a coefficient of 0.83, $P<0.000006$.

Fine needle aspiration is one of the most reliable methods for the diagnosis of carcinoma of the breast. In the hands of experts, in fact, it proves to have extremely high sensitivity, specificity, positive predictive value and negative predictive value (Magdelenat *et al.*, 1986). The technique offers several advantages; it is atraumatic and does not require the use of local anaesthesia; it can be repeated and is well-accepted by patients, which means that it may be used in sequential studies and for follow-up. Fine needle aspiration may be performed on deep organs under echographic or computerised tomographic control.

More recently, the material obtained by means of fine needle aspiration has been used for the determination of oestrogen receptors (ER) and progesterone receptors (PR) by means of the traditional biochemical method (Benyahia *et al.*, 1982; Merle *et al.*, 1985, 1986). Compared to the results of determination performed on excised specimens, the average accuracy of this technique is of 90%, but it is only possible where the DNA concentration exceeds $20 \mu m\, ml^{-1}$ of cytosol, that is, when more than 1,000,000 cells are present (Benyahia *et al.*, 1982; Merle *et al.*, 1985, 1986). Furthermore, it does not offer any technical advantages compared to the traditional method, since it needs the same apparatus and cannot therefore be easily performed in small hospitals or laboratories.

The production of monoclonal antibodies which are specific for the receptors and the development of the immunoperoxidase technique (Flowers *et al.*, 1986; Green & Jensen, 1982) have made it possible to analyse the ER contents in cytological preparations containing a very small number of cells.

We have used monoclonal antibodies for the determination of the ER on smears from fine needle aspirates obtained from breast carcinomas.

### Materials and methods

From February 1987 until February 1988, 50 patients aged from 29 to 74 years, with an average of 59.2, affected by primary operable breast carcinoma, 20 at stage I and 30 at stage II, underwent fine needle aspiration with cytological examination and assessment of the ER contents. After surgery, the ER and PR contents were examined on the excised specimens by means of the traditional biochemical method (Castagnetta *et al.*, 1983; Clark & Peck, 1979; Leake *et al.*, 1979) (dextran-coated charcoal adsorption assay). Four of the 50 cases could not be assessed because there were no cells in the cytological smears.

Correspondence: A. Marrazzo.
Received 5 June 1988, and in revised form, 7 November 1988.

### *Immunocytochemical analysis*

The cytological sampling was performed with 23 gauge needles attached to $5\,cm^3$ syringes, passed four or five times in various directions in the neoplasia. The material thus obtained was then smeared on to slides which had been pretreated with a protenaceous solution containing an anti-microbial substance. Two slides were prepared for each patient, one with anti-ER antibodies and the other as a control.

For fixation, the frozen slides were immersed in 3.6% formol/phosphate buffered saline (PBS) at room temperature for 15 min and then washed twice in PBS for a total of 10 min. They were then placed in absolute methanol ($-20°C$) for 2 min, washed again in PBS at room temperature for 5 min and stored in a cold ($-20°C$) preparation of 250 ml glycerol + 42.8 g saccharose + 0.33 g chloride of magnesium with the addition of up to 500 ml of PBS serum.

For the staining procedure we used the H222 Monoclonal Antibody ER Assay Kit produced by Abbott Laboratories, no. 3087-19, using one drop of solution per slide.

### *Determination of the receptors*

The traditional biochemical determination is expressed in femtomol per milligram of cytosol. The immunocytochemical determination was performed by means of the semi-quantitative technique. The receptors are located inside the nuclei and look brown if positive and light grey if negative. The following parameters were taken into consideration: intensity of staining, divided into (1) slightly, (2) moderately and (3) intensely stained; percentage of epithelial cells with positive receptors compared to the total number of epithelial cells.

The receptor value is calculated by multiplying the staining intensity by the percentage of positive epithelial cells. The scale ranges from 0 to 300. The threshold value for positivity for the biochemical method is $10\,fmol\,mg^{-1}$ of cytosol.

### *Data analysis*

The validity of the immunocytochemical method was assessed by calculating the sensitivity, the specificity, the positive predictive value, the negative predictive value and the accuracy. These are calculated as follows:

$$\text{sensitivity} = \frac{\text{true positives (TP)}}{\text{true positives + false negatives (FN)}}$$

$$\text{specificity} = \frac{\text{true negatives (TN)}}{\text{true negatives + false positives (FP)}}$$

$$\text{positive predictive value} = \frac{TP}{TP+FP}$$

$$\text{negative predictive value} = \frac{TN}{TN+FN}$$

$$\text{accuracy} = \frac{TP+TN}{TP+TN+FP+FN}.$$

A comparison was made with the results obtained with the semiquantitative and biochemical techniques by means of the correlation coefficient and linear regression, with the values expressed on a logarithmic scale.

### Results

Of the 46 evaluable cases, the biochemical method showed an ER concentration which was equal to or more than 10 fmol $mg^{-1}$ of cytosol in 29 cases, which were therefore classified as positive. In 17 cases the ER concentration was less than 10 fmol $mg^{-1}$ of cytosol and these were considered as negative. Twenty-nine positive cases also gave a positive result with the immunocytochemical method, while two of the 17 negative cases also showed as positive with this technique (Table I). One of these two positives concerned a premenopausal patient; the other case was a post-menopausal patient with 7.5 fmol $mg^{-1}$ of cytosol ER and 1331.5 fmol $mg^{-1}$ of cytosol PR. The immunocytochemical method showed 100% sensitivity, 88.2% specificity, 93.5% positive predictive value, 100% negative predictive value and 95.6% accuracy.

Determination of ERs: the accuracy of the immunocytological method compared with the biochemical method in determining ERs was: semiquantitative value $>20$; sensitivity 100%; specificity 88.2%; positive predictive value 93.5%; negative predictive value 100%.

The correlation between the semiquantitative immunocytochemical and quantitative biochemical methods, calculated by means of linear regression, showed the equation $y = 0.27 + 0.78$, $P < 0.000006$, and a correlation coefficient of 0.83, $P < 0.000006$ (Figure 1).

### Discussion

The determination of the hormone receptors plays an extremely important role in the therapeutic management of breast carcinoma since it can be used as a guide for the treatment protocol of choice. ER positive tumours in fact respond to hormone therapy in 60% of the cases, while ER negative tumours show a response in less than 10% (Osborne & McGuire, 1978).

The traditional method requires that the receptor assay be made biochemically on excised specimens using the radioligand technique. For this method, however, from 0.5 to 1 g of tissue is necessary (Castagnetta *et al.*, 1983) plus sophisticated apparatus which may not always be available in smaller hospitals.

Earlier diagnosis of carcinoma of the breast, together with the larger number of investigations regarding the biology of

**Figure 1** Correlation of the logarithms of the ER values between the biochemical (concentration of ERs expressed as fmol $mg^{-1}$) and. immunocytochemical (% positive ERICA cells × staining intensity) methods.

the neoplasia, such as ER, cellular kinetics and DNA, now mean that in some cases the quantity of tumoral tissue may not be enough for histological diagnosis and biological characterisation. Furthermore, patients with inoperable tumours refuse to undergo surgery, and in those with distant metastases of deep organs the tumoral tissue may be difficult to get at.

Fine needle cytology is a not very invasive, cheap technique, which is extremely useful for the sampling of a number of tumoral cells sufficient for the biological characterisation of the tumour, for example, by means of the determination of the ER. With the traditional biochemical method this latter requires a quantity of DNA of at least 20 $\mu g\,ml^{-1}$ of cytosol, that is, containing 1,000,000 cells (Benyahia *et al.*, 1982; Merle *et al.*, 1985, 1986).

The advent of monoclonal antibodies has proved extremely important for the determination of the ER in several studies, most of which have been performed on tiny slices of tumoral tissue by means of the enzyme–immunoassay technique. Compared to the classical biochemical method, these studies have shown an average of 90% sensibility, 82.3% specificity, 86.7% positive predictive value (PPV) and 87.2% negative predictive value (NPV) (Pertschuck *et al.*, 1985; McCarty *et al.*, 1985; King *et al.*, 1985; Shimada *et al.*, 1985). Furthermore, the predictability of the response to hormone therapy would seem to be higher compared to the biochemical method (Pertschuck *et al.*, 1985; McCarty *et al.*, 1985).

Enzyme-immunoassay with monoclonal antibodies has also been assessed on fine needle aspirates. Magdelenat *et al.* (1986) report that it is completely reliable, with a correlation coefficient when the technique is performed on excised specimens of 0.97. Moreover, it has a higher sensitivity than the biochemical method performed on fine needle aspirates,

**Table I** Determination of ERs, comparison between immunocytological method on fine needle aspirates and biochemical method on excised specimens

| | *Authors* | *Pos. value* | *Neg. value* | *False pos.* | *False neg.* |
|---|---|---|---|---|---|
| Crawford *et al.* (1985) | 20 | 23 | 2 | 1 | |
| Flowers *et al.* (1986) | 12 | 16 | 2 | 3 | |
| Azavedo *et al.* (1986) | 20 | 8 | 3 | 0 | |
| Our results | 29 | 15 | 2 | 0 | |
| Total | 81 | 62 | 9 | 4 | |
| | Sens. | Spec. | PPV | NPV | Acc. |
| | 95.2% | 87.3% | 91% | 93.9% | 91% |

and is possible on samples containing 10 μg of DNA per ml of cytosol (Magdelenat *et al.*, 1986).

The immunocytological technique with monoclonal antibodies performed on fine needle aspirates offers the advantage of being easier and more simple to perform and of needing no special apparatus. It shows 90% accuracy, 92.8% sensitivity, 87.03% specificity, 88.1% PPV and 92.1% NPV. Crawford *et al.* (1985) report 20 true positives, 22 true negatives, two false positives and one false negative, while Flowers *et al.* (1986) obtained 12 true positives, 16 true negatives, two false positives and three false negatives, and Azavedo *et al.* (1986) 20 true positives, eight true negatives, three false positives and no false negatives (Table I).

In our own series we did not obtain any probably untrue negatives, while one of the two discordant positives showed an extremely high concentration of PR. The method also offers a semiquantitative determination of the ER contents with an acceptable correlation (Azavedo *et al.*, 1986; Flowers *et al.*, 1986; Crawford *et al.*, 1985), and we ourselves found a significant correlation with a coefficient of 0.83. It has been demonstrated that the ER contents may vary from one area of the carcinoma to another; cell samples aspirated in different directions within the tumour make it possible to obtain a more complete picture of the receptor status of the neoplasia. The material examined must contain a sufficient quantity of tumoral tissue to make an accurate identification of ER negative tumours, since the level of the receptors depends on the cellularity of the carcinoma. A study assessing the quantity of tumoral tissue reports that in 24% of the ER negative cases there was a quantity of less than 10%, which is not sufficient for an accurate analysis (Steele *et al.*, 1987). The determination on smears prepared from fine needle aspirates makes it possible to make a simultaneous assessment of the cellular quantity.

At the present time the biochemical method is used when the patient must undergo surgery; in cases of distant metastases in deep organs, the immunocytochemical technique may give excellent information regarding the ER status, and may also be extremely efficient for studying the sequential determination of the ER.

### References

AZAVEDO, E., BARAL, E. & SKOOG, L. (1986). Immunohistochemical analysis of estrogen receptors in cells obtained by fine needle aspiration from human carcinomas. *Anticancer Res.*, **6**, 263.

BENYAHIA, B., MAGDELENAT, H., ZAJDELA, A. & WILCOQ, J.R. (1982). Ponction-aspiration l'aguille fine et dosages des recepteurs d'oestrogenes dans le cancer du sein. *Bull. Cancer*, **69**, 456.

CASTAGNETTA, L., LO CASTO, N., MERCADANTE, T. *et al.* (1983). Intra-tumoral variation of oestrogen receptor status in endometrial cancer. *Br. J. Cancer*, **47**, 261.

CLARK, S.H. & PECK, E.J. JR. (1979). *Female Sex Steroid Receptors and Function.* Springer-Verlag: Berlin.

CRAWFORD, J.L., LOPE-PIHIE, A., COWAN, S., GEORGE, W.D. & LEAKE, R.E. (1985). Preoperative determination of oestrogen receptor status in breast cancer by immunocytochemical staining of fine needle aspirates. *Br. J. Surg.*, **72**, 991.

FLOWERS, J.L., COX, E.B., GEISINGER, K.R. *et al.* (1986). Use of monoclonal antiestrogen receptor antibody to evaluate estrogen receptor content in fine needle aspiration breast biopsies. *Ann. Surg.*, **203**, 250.

GREEN, G.L. & JENSEN, E.V. (1982). Monoclonal antibodies as probes for estrogen receptor detection and characterization. *J. Steroid Biochem.*, **16**, 353.

KING, W.J., DE SOMBRE, E.R., JENSEN, E.V. & GREEN, G.L. (1985). Comparison of immunocytochemical and steroid-binding assays for estrogen receptors in human breast tumor. *Cancer Res.*, **45**, 393.

LEAKE, R.E., LAING, L. & SMITH, D.C. (1979). *Steroid Receptor Assay in Human Breast Tumours: Methodological and Clinical Aspects*, King, R.S.B. (ed) p. 73. Alpha Omega Alpha Publishing: Cardiff.

LECLERQ, G., HEUSON, J.L., SHOENFELD, R., MATTHEIM, W.H. & TAGNON, H.J. (1973). Estrogen receptors in human breast cancer. *Eur. J. Cancer*, **9**, 665.

MAGDELENAT, H., MERLE, S. & ZAJDELA, A. (1986). Enzymo-immunoassay of estrogen receptors in fine needle aspirates of breast tumors. *Cancer Res.*, suppl. 46, 4265.

MARRAZZO, A., LI VOTI, G. & DI CUONZO, G. (1979). Sensitivity and specificity of aspiration cytology in malignant breast diseases. *Senologia*, **4**, 217.

McCARTY, K.S., MILLER, L.S. & COX, E.R. (1985). Estrogen receptor analyses. *Arch. Pathol. Lab. Med.*, **109**, 716.

MERLE, S., ZAJDELA, A. & MAGDELENAT, H. (1985). Progesterone receptor assay in fine needle aspirates of breast tumors. *Acta Cytol.*, **29**, 496.

MERLE, S., ZAJDELA, A., MAGDELENAT, H. *et al.* (1986). Applications of immunopathology and immunology to diagnostic cytology. International Congress of Cytology, Brussels.

OSBORNE, C.K. & McGUIRE, W.L. (1978). Current use of steroid hormone receptor assay in the treatment of breast cancer. *Surg. Clin. N. Am.*, **58**, 777.

PERTSCHUK, L.P., EISENBERG, K.B., CARTER, A.K. *et al.* (1985). Immunohistologic localization of estrogen receptors in breast cancer with monoclonal antibodies. *Cancer*, **55**, 1513.

SHIMADA, A., KIMURA, S., ABE, K. *et al.* (1985). Immunocytochemical staining of estrogen receptors in paraffin sections of human breast cancer by use of monoclonal antibody: comparison with that in frozen sections. *Proc. Natl Acad. Sci. USA*, **82**, 4803.

STEELE, J.C., HAWKINS, R.A., ANDERSON, T.J. & FORREST, A.P.M. (1987). The relevance of control histology in oestrogen receptor estimation. *Br. J. Cancer.*, **56**, 617.